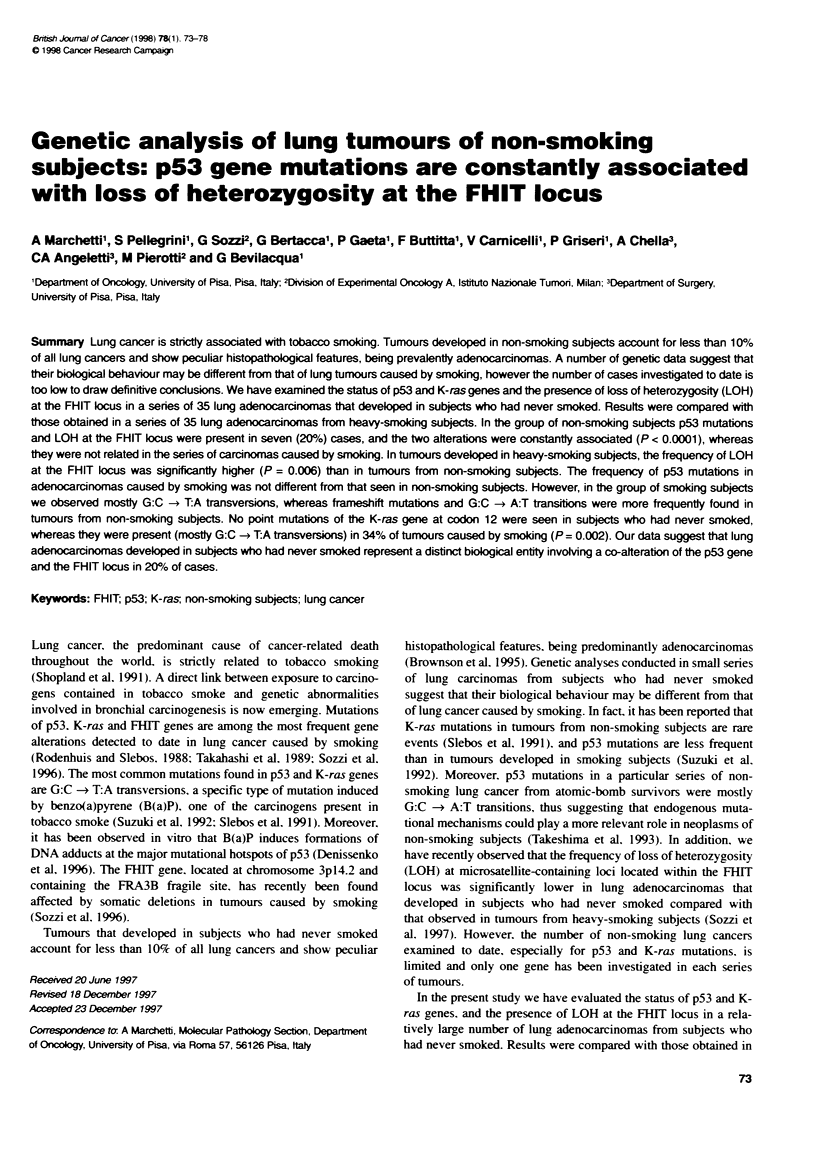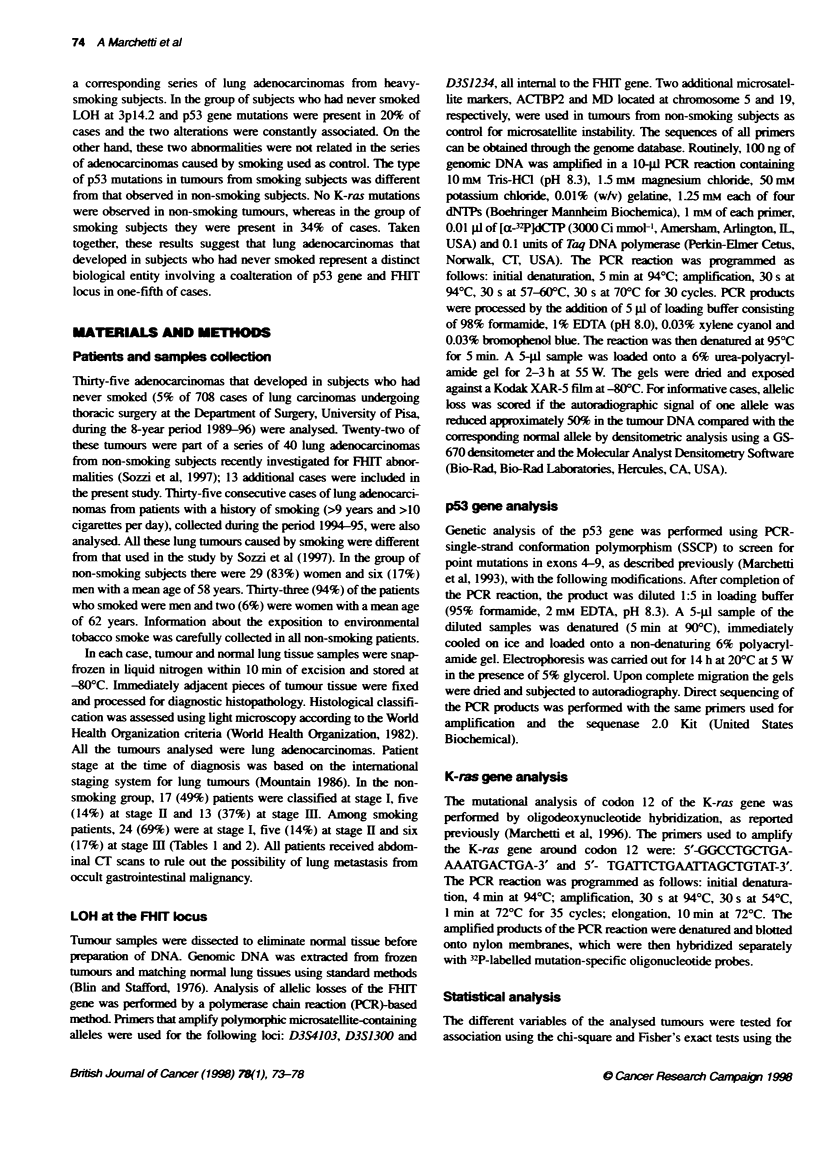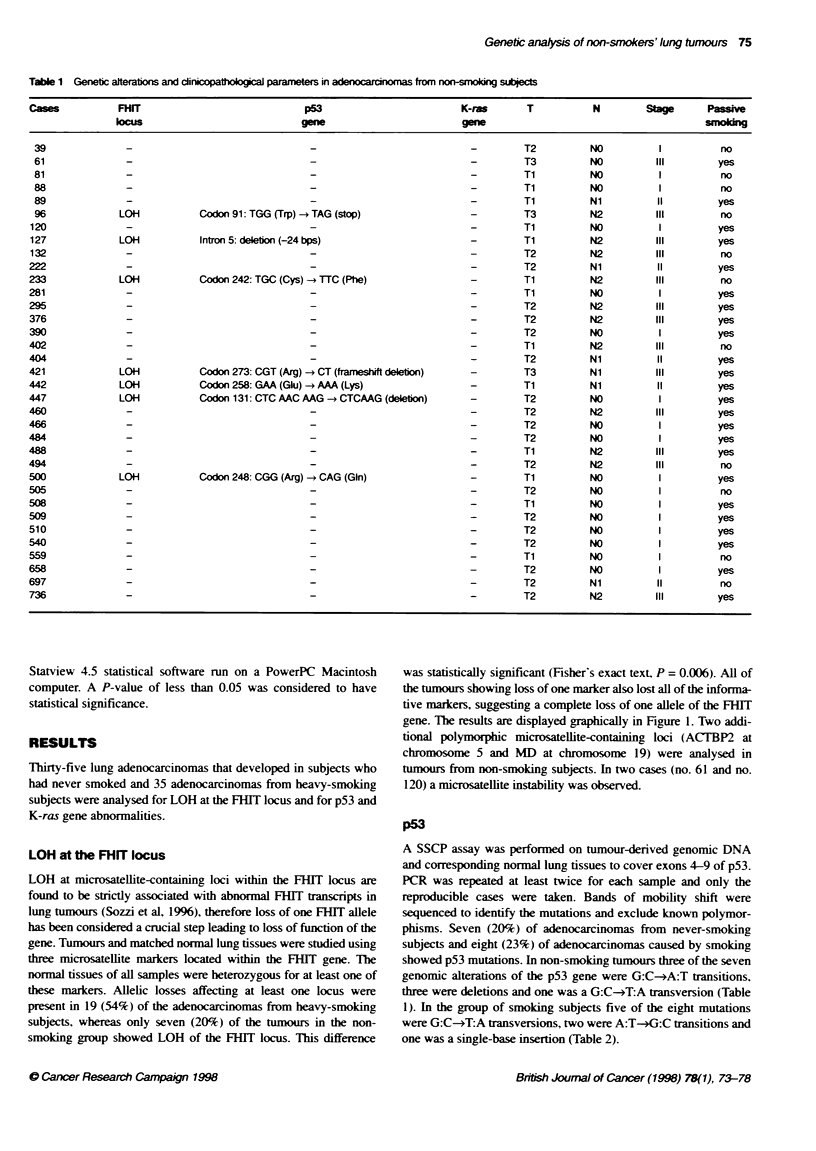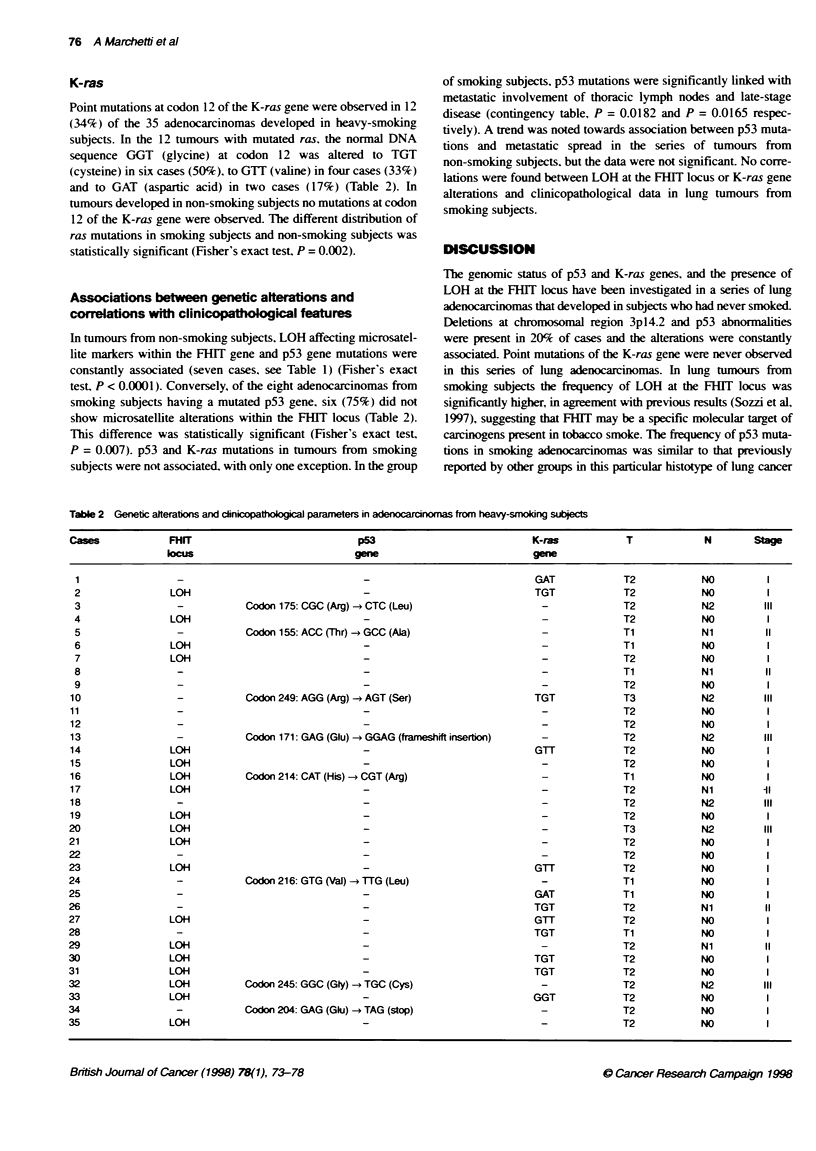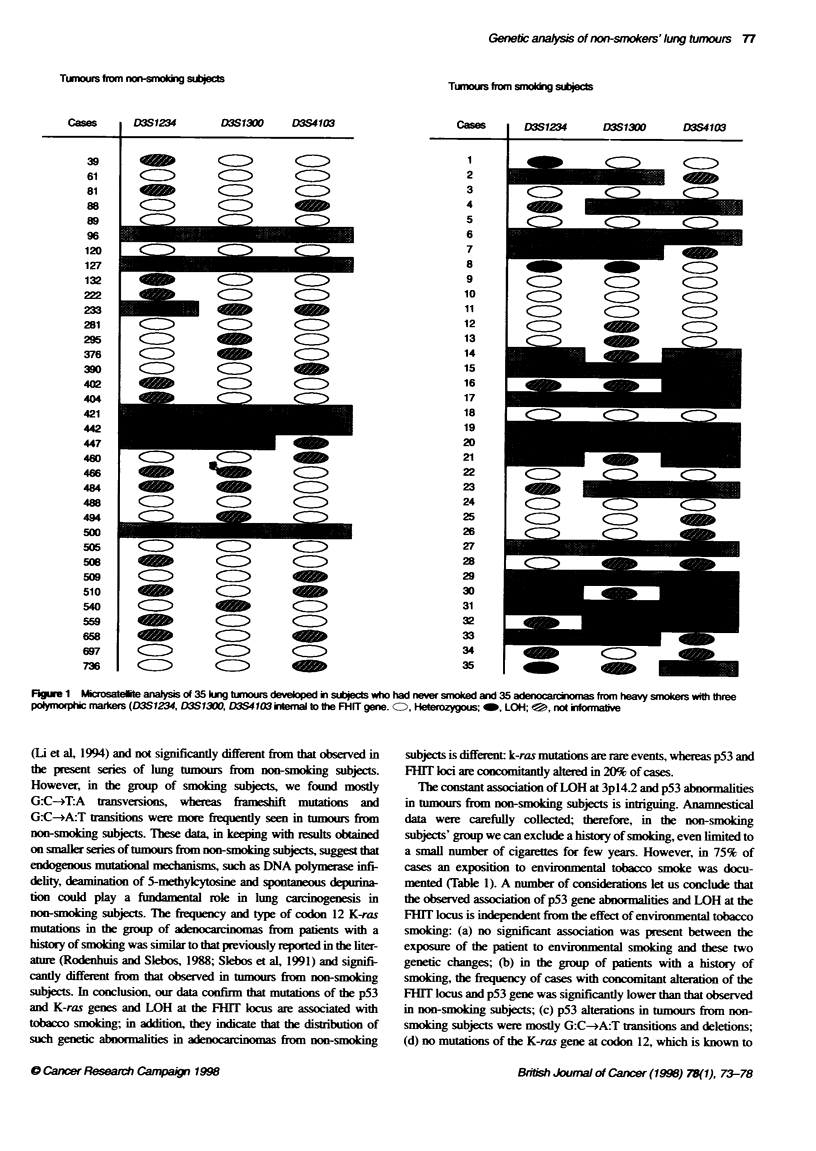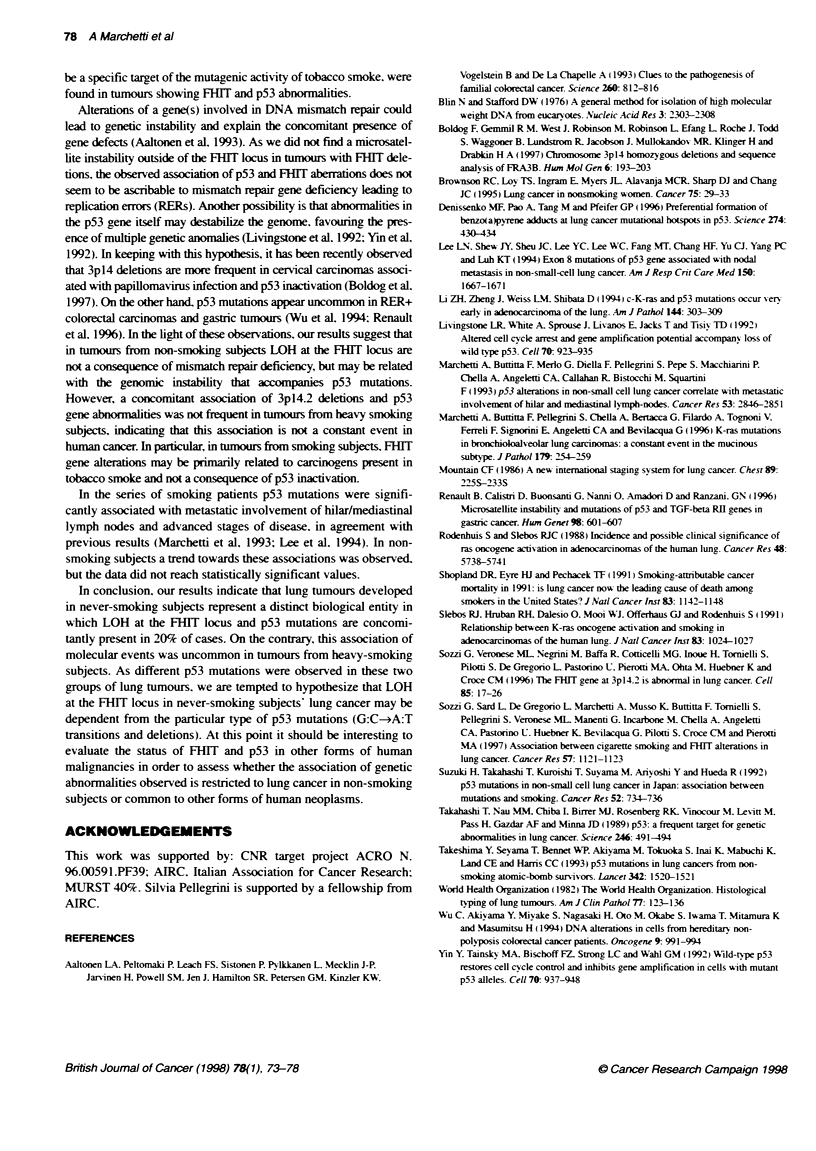# Genetic analysis of lung tumours of non-smoking subjects: p53 gene mutations are constantly associated with loss of heterozygosity at the FHIT locus.

**DOI:** 10.1038/bjc.1998.445

**Published:** 1998-07

**Authors:** A. Marchetti, S. Pellegrini, G. Sozzi, G. Bertacca, P. Gaeta, F. Buttitta, V. Carnicelli, P. Griseri, A. Chella, C. A. Angeletti, M. Pierotti, G. Bevilacqua

**Affiliations:** Department of Oncology, University of Pisa, Italy.

## Abstract

Lung cancer is strictly associated with tobacco smoking. Tumours developed in non-smoking subjects account for less than 10% of all lung cancers and show peculiar histopathological features, being prevalently adenocarcinomas. A number of genetic data suggest that their biological behaviour may be different from that of lung tumours caused by smoking, however the number of cases investigated to date is too low to draw definitive conclusions. We have examined the status of p53 and K-ras genes and the presence of loss of heterozygosity (LOH) at the FHIT locus in a series of 35 lung adenocarcinomas that developed in subjects who had never smoked. Results were compared with those obtained in a series of 35 lung adenocarcinomas from heavy-smoking subjects. In the group of non-smoking subjects p53 mutations and LOH at the FHIT locus were present in seven (20%) cases, and the two alterations were constantly associated (P < 0.0001), whereas they were not related in the series of carcinomas caused by smoking. In tumours developed in heavy-smoking subjects, the frequency of LOH at the FHIT locus was significantly higher (P = 0.006) than in tumours from non-smoking subjects. The frequency of p53 mutations in adenocarcinomas caused by smoking was not different from that seen in non-smoking subjects. However, in the group of smoking subjects we observed mostly G:C --> T:A transversions, whereas frameshift mutations and G:C --> A:T transitions were more frequently found in tumours from non-smoking subjects. No point mutations of the K-ras gene at codon 12 were seen in subjects who had never smoked, whereas they were present (mostly G:C --> T:A transversions) in 34% of tumours caused by smoking (P = 0.002). Our data suggest that lung adenocarcinomas developed in subjects who had never smoked represent a distinct biological entity involving a co-alteration of the p53 gene and the FHIT locus in 20% of cases.


British Journal of Cancer (1998) 78(1), 73–78
© 1998 Cancer Research Campaign

### Genetic analysis of lung tumours of non-smoking subjects: p53 gene mutations are constantly associated with loss of heterozygosity at the FHIT locus

A Marchetti[1], S Pellegrini[1], G Sozzi[2], G Bertacca[1], P Gaeta[1], F Buttitta[1], V Carnicelli[1], P Griseri[1], A Chella[3], CA Angeletti[3], M Pierotti[2] and G Bevilacqua[1]

[1]Department of Oncology, University of Pisa, Pisa, Italy; [2]Division of Experimental Oncology A, Istituto Nazionale Tumori, Milan; [3]Department of Surgery, University of Pisa, Pisa, Italy

**Summary** Lung cancer is strictly associated with tobacco smoking. Tumours developed in non-smoking subjects account for less than 10% of all lung cancers and show peculiar histopathological features, being prevalently adenocarcinomas. A number of genetic data suggest that their biological behaviour may be different from that of lung tumours caused by smoking, however the number of cases investigated to date is too low to draw definitive conclusions. We have examined the status of p53 and K-*ras* genes and the presence of loss of heterozygosity (LOH) at the FHIT locus in a series of 35 lung adenocarcinomas that developed in subjects who had never smoked. Results were compared with those obtained in a series of 35 lung adenocarcinomas from heavy-smoking subjects. In the group of non-smoking subjects p53 mutations and LOH at the FHIT locus were present in seven (20%) cases, and the two alterations were constantly associated ($P < 0.0001$), whereas they were not related in the series of carcinomas caused by smoking. In tumours developed in heavy-smoking subjects, the frequency of LOH at the FHIT locus was significantly higher ($P = 0.006$) than in tumours from non-smoking subjects. The frequency of p53 mutations in adenocarcinomas caused by smoking was not different from that seen in non-smoking subjects. However, in the group of smoking subjects we observed mostly G:C → T:A transversions, whereas frameshift mutations and G:C → A:T transitions were more frequently found in tumours from non-smoking subjects. No point mutations of the K-*ras* gene at codon 12 were seen in subjects who had never smoked, whereas they were present (mostly G:C → T:A transversions) in 34% of tumours caused by smoking ($P = 0.002$). Our data suggest that lung adenocarcinomas developed in subjects who had never smoked represent a distinct biological entity involving a co-alteration of the p53 gene and the FHIT locus in 20% of cases.

**Keywords:** FHIT; p53; K-*ras*; non-smoking subjects; lung cancer

Lung cancer, the predominant cause of cancer-related death throughout the world, is strictly related to tobacco smoking (Shopland et al, 1991). A direct link between exposure to carcinogens contained in tobacco smoke and genetic abnormalities involved in bronchial carcinogenesis is now emerging. Mutations of p53, K-*ras* and FHIT genes are among the most frequent gene alterations detected to date in lung cancer caused by smoking (Rodenhuis and Slebos, 1988; Takahashi et al, 1989; Sozzi et al, 1996). The most common mutations found in p53 and K-*ras* genes are G:C → T:A transversions, a specific type of mutation induced by benzo(a)pyrene (B(a)P), one of the carcinogens present in tobacco smoke (Suzuki et al, 1992; Slebos et al, 1991). Moreover, it has been observed in vitro that B(a)P induces formations of DNA adducts at the major mutational hotspots of p53 (Denissenko et al, 1996). The FHIT gene, located at chromosome 3p14.2 and containing the FRA3B fragile site, has recently been found affected by somatic deletions in tumours caused by smoking (Sozzi et al, 1996).

Tumours that developed in subjects who had never smoked account for less than 10% of all lung cancers and show peculiar histopathological features, being predominantly adenocarcinomas (Brownson et al, 1995). Genetic analyses conducted in small series of lung carcinomas from subjects who had never smoked suggest that their biological behaviour may be different from that of lung cancer caused by smoking. In fact, it has been reported that K-*ras* mutations in tumours from non-smoking subjects are rare events (Slebos et al, 1991), and p53 mutations are less frequent than in tumours developed in smoking subjects (Suzuki et al, 1992). Moreover, p53 mutations in a particular series of non-smoking lung cancer from atomic-bomb survivors were mostly G:C → A:T transitions, thus suggesting that endogenous mutational mechanisms could play a more relevant role in neoplasms of non-smoking subjects (Takeshima et al, 1993). In addition, we have recently observed that the frequency of loss of heterozygosity (LOH) at microsatellite-containing loci located within the FHIT locus was significantly lower in lung adenocarcinomas that developed in subjects who had never smoked compared with that observed in tumours from heavy-smoking subjects (Sozzi et al, 1997). However, the number of non-smoking lung cancers examined to date, especially for p53 and K-*ras* mutations, is limited and only one gene has been investigated in each series of tumours.

In the present study we have evaluated the status of p53 and K-*ras* genes, and the presence of LOH at the FHIT locus in a relatively large number of lung adenocarcinomas from subjects who had never smoked. Results were compared with those obtained in

*Received 20 June 1997*
*Revised 18 December 1997*
*Accepted 23 December 1997*

*Correspondence to:* A Marchetti, Molecular Pathology Section, Department of Oncology, University of Pisa, via Roma 57, 56126 Pisa, Italy a corresponding series of lung adenocarcinomas from heavy-smoking subjects. In the group of subjects who had never smoked LOH at 3p14.2 and p53 gene mutations were present in 20% of cases and the two alterations were constantly associated. On the other hand, these two abnormalities were not related in the series of adenocarcinomas caused by smoking used as control. The type of p53 mutations in tumours from smoking subjects was different from that observed in non-smoking subjects. No K-*ras* mutations were observed in non-smoking tumours, whereas in the group of smoking subjects they were present in 34% of cases. Taken together, these results suggest that lung adenocarcinomas that developed in subjects who had never smoked represent a distinct biological entity involving a coalteration of p53 gene and FHIT locus in one-fifth of cases.

### MATERIALS AND METHODS

### Patients and samples collection

Thirty-five adenocarcinomas that developed in subjects who had never smoked (5% of 708 cases of lung carcinomas undergoing thoracic surgery at the Department of Surgery, University of Pisa, during the 8-year period 1989–96) were analysed. Twenty-two of these tumours were part of a series of 40 lung adenocarcinomas from non-smoking subjects recently investigated for FHIT abnormalities (Sozzi et al, 1997); 13 additional cases were included in the present study. Thirty-five consecutive cases of lung adenocarcinomas from patients with a history of smoking (>9 years and >10 cigarettes per day), collected during the period 1994–95, were also analysed. All these lung tumours caused by smoking were different from that used in the study by Sozzi et al (1997). In the group of non-smoking subjects there were 29 (83%) women and six (17%) men with a mean age of 58 years. Thirty-three (94%) of the patients who smoked were men and two (6%) were women with a mean age of 62 years. Information about the exposition to environmental tobacco smoke was carefully collected in all non-smoking patients.

In each case, tumour and normal lung tissue samples were snap-frozen in liquid nitrogen within 10 min of excision and stored at −80°C. Immediately adjacent pieces of tumour tissue were fixed and processed for diagnostic histopathology. Histological classification was assessed using light microscopy according to the World Health Organization criteria (World Health Organization, 1982). All the tumours analysed were lung adenocarcinomas. Patient stage at the time of diagnosis was based on the international staging system for lung tumours (Mountain 1986). In the non-smoking group, 17 (49%) patients were classified at stage I, five (14%) at stage II and 13 (37%) at stage III. Among smoking patients, 24 (69%) were at stage I, five (14%) at stage II and six (17%) at stage III (Tables 1 and 2). All patients received abdominal CT scans to rule out the possibility of lung metastasis from occult gastrointestinal malignancy.

### LOH at the FHIT locus

Tumour samples were dissected to eliminate normal tissue before preparation of DNA. Genomic DNA was extracted from frozen tumours and matching normal lung tissues using standard methods (Blin and Stafford, 1976). Analysis of allelic losses of the FHIT gene was performed by a polymerase chain reaction (PCR)-based method. Primers that amplify polymorphic microsatellite-containing alleles were used for the following loci: *D3S4103*, *D3S1300* and *D3S1234*, all internal to the FHIT gene. Two additional microsatellite markers, ACTBP2 and MD located at chromosome 5 and 19, respectively, were used in tumours from non-smoking subjects as control for microsatellite instability. The sequences of all primers can be obtained through the genome database. Routinely, 100 ng of genomic DNA was amplified in a 10-μl PCR reaction containing 10 mM Tris-HCl (pH 8.3), 1.5 mM magnesium chloride, 50 mM potassium chloride, 0.01% (w/v) gelatine, 1.25 mM each of four dNTPs (Boehringer Mannheim Biochemica), 1 mM of each primer, 0.01 μl of [α-$^{32}$P]dCTP (3000 Ci mmol$^{-1}$, Amersham, Arlington, IL, USA) and 0.1 units of *Taq* DNA polymerase (Perkin-Elmer Cetus, Norwalk, CT, USA). The PCR reaction was programmed as follows: initial denaturation, 5 min at 94°C; amplification, 30 s at 94°C, 30 s at 57–60°C, 30 s at 70°C for 30 cycles. PCR products were processed by the addition of 5 μl of loading buffer consisting of 98% formamide, 1% EDTA (pH 8.0), 0.03% xylene cyanol and 0.03% bromophenol blue. The reaction was then denatured at 95°C for 5 min. A 5-μl sample was loaded onto a 6% urea-polyacrylamide gel for 2–3 h at 55 W. The gels were dried and exposed against a Kodak XAR-5 film at −80°C. For informative cases, allelic loss was scored if the autoradiographic signal of one allele was reduced approximately 50% in the tumour DNA compared with the corresponding normal allele by densitometric analysis using a GS-670 densitometer and the Molecular Analyst Densitometry Software (Bio-Rad, Bio-Rad Laboratories, Hercules, CA, USA).

### p53 gene analysis

Genetic analysis of the p53 gene was performed using PCR-single-strand conformation polymorphism (SSCP) to screen for point mutations in exons 4–9, as described previously (Marchetti et al, 1993), with the following modifications. After completion of the PCR reaction, the product was diluted 1:5 in loading buffer (95% formamide, 2 mM EDTA, pH 8.3). A 5-μl sample of the diluted samples was denatured (5 min at 90°C), immediately cooled on ice and loaded onto a non-denaturing 6% polyacrylamide gel. Electrophoresis was carried out for 14 h at 20°C at 5 W in the presence of 5% glycerol. Upon complete migration the gels were dried and subjected to autoradiography. Direct sequencing of the PCR products was performed with the same primers used for amplification and the sequenase 2.0 Kit (United States Biochemical).

### K-*ras* gene analysis

The mutational analysis of codon 12 of the K-*ras* gene was performed by oligodeoxynucleotide hybridization, as reported previously (Marchetti et al, 1996). The primers used to amplify the K-*ras* gene around codon 12 were: 5′-GGCCTGCTGA-AAATGACTGA-3′ and 5′- TGATTCTGAATTAGCTGTAT-3′. The PCR reaction was programmed as follows: initial denaturation, 4 min at 94°C; amplification, 30 s at 94°C, 30 s at 54°C, 1 min at 72°C for 35 cycles; elongation, 10 min at 72°C. The amplified products of the PCR reaction were denatured and blotted onto nylon membranes, which were then hybridized separately with $^{32}$P-labelled mutation-specific oligonucleotide probes.

### Statistical analysis

The different variables of the analysed tumours were tested for association using the chi-square and Fisher's exact tests using the

© Cancer Research Campaign 1998

**Table 1** Genetic alterations and clinicopathological parameters in adenocarcinomas from non-smoking subjects

| Cases | FHIT locus | p53 gene | K-*ras* gene | T | N | Stage | Passive smoking |
|---|---|---|---|---|---|---|---|
| 39 | – | – | – | T2 | N0 | I | no |
| 61 | – | – | – | T3 | N0 | III | yes |
| 81 | – | – | – | T1 | N0 | I | no |
| 88 | – | – | – | T1 | N0 | I | no |
| 89 | – | – | – | T1 | N1 | II | yes |
| 96 | LOH | Codon 91: TGG (Trp) → TAG (stop) | – | T3 | N2 | III | no |
| 120 | – | – | – | T1 | N0 | I | yes |
| 127 | LOH | Intron 5: deletion (–24 bps) | – | T1 | N2 | III | yes |
| 132 | – | – | – | T2 | N2 | III | no |
| 222 | – | – | – | T2 | N1 | II | yes |
| 233 | LOH | Codon 242: TGC (Cys) → TTC (Phe) | – | T1 | N2 | III | no |
| 281 | – | – | – | T1 | N0 | I | yes |
| 295 | – | – | – | T2 | N2 | III | yes |
| 376 | – | – | – | T2 | N2 | III | yes |
| 390 | – | – | – | T2 | N0 | I | yes |
| 402 | – | – | – | T1 | N2 | III | no |
| 404 | – | – | – | T2 | N1 | II | yes |
| 421 | LOH | Codon 273: CGT (Arg) → CT (frameshift deletion) | – | T3 | N1 | III | yes |
| 442 | LOH | Codon 258: GAA (Glu) → AAA (Lys) | – | T1 | N1 | II | yes |
| 447 | LOH | Codon 131: CTC AAC AAG → CTCAAG (deletion) | – | T2 | N0 | I | yes |
| 460 | – | – | – | T2 | N2 | III | yes |
| 466 | – | – | – | T2 | N0 | I | yes |
| 484 | – | – | – | T2 | N0 | I | yes |
| 488 | – | – | – | T1 | N2 | III | yes |
| 494 | – | – | – | T2 | N2 | III | no |
| 500 | LOH | Codon 248: CGG (Arg) → CAG (Gln) | – | T1 | N0 | I | yes |
| 505 | – | – | – | T2 | N0 | I | no |
| 508 | – | – | – | T1 | N0 | I | yes |
| 509 | – | – | – | T2 | N0 | I | yes |
| 510 | – | – | – | T2 | N0 | I | yes |
| 540 | – | – | – | T2 | N0 | I | yes |
| 559 | – | – | – | T1 | N0 | I | no |
| 658 | – | – | – | T2 | N0 | I | yes |
| 697 | – | – | – | T2 | N1 | II | no |
| 736 | – | – | – | T2 | N2 | III | yes |

Statview 4.5 statistical software run on a PowerPC Macintosh computer. A $P$-value of less than 0.05 was considered to have statistical significance.

### RESULTS

Thirty-five lung adenocarcinomas that developed in subjects who had never smoked and 35 adenocarcinomas from heavy-smoking subjects were analysed for LOH at the FHIT locus and for p53 and K-*ras* gene abnormalities.

### LOH at the FHIT locus

LOH at microsatellite-containing loci within the FHIT locus are found to be strictly associated with abnormal FHIT transcripts in lung tumours (Sozzi et al, 1996), therefore loss of one FHIT allele has been considered a crucial step leading to loss of function of the gene. Tumours and matched normal lung tissues were studied using three microsatellite markers located within the FHIT gene. The normal tissues of all samples were heterozygous for at least one of these markers. Allelic losses affecting at least one locus were present in 19 (54%) of the adenocarcinomas from heavy-smoking subjects, whereas only seven (20%) of the tumours in the non-smoking group showed LOH of the FHIT locus. This difference was statistically significant (Fisher's exact text, $P = 0.006$). All of the tumours showing loss of one marker also lost all of the informative markers, suggesting a complete loss of one allele of the FHIT gene. The results are displayed graphically in Figure 1. Two additional polymorphic microsatellite-containing loci (ACTBP2 at chromosome 5 and MD at chromosome 19) were analysed in tumours from non-smoking subjects. In two cases (no. 61 and no. 120) a microsatellite instability was observed.

### p53

A SSCP assay was performed on tumour-derived genomic DNA and corresponding normal lung tissues to cover exons 4–9 of p53. PCR was repeated at least twice for each sample and only the reproducible cases were taken. Bands of mobility shift were sequenced to identify the mutations and exclude known polymorphisms. Seven (20%) of adenocarcinomas from never-smoking subjects and eight (23%) of adenocarcinomas caused by smoking showed p53 mutations. In non-smoking tumours three of the seven genomic alterations of the p53 gene were G:C→A:T transitions, three were deletions and one was a G:C→T:A transversion (Table 1). In the group of smoking subjects five of the eight mutations were G:C→T:A transversions, two were A:T→G:C transitions and one was a single-base insertion (Table 2).

© *Cancer Research Campaign 1998* 

### K-*ras*

Point mutations at codon 12 of the K-*ras* gene were observed in 12 (34%) of the 35 adenocarcinomas developed in heavy-smoking subjects. In the 12 tumours with mutated *ras*, the normal DNA sequence GGT (glycine) at codon 12 was altered to TGT (cysteine) in six cases (50%), to GTT (valine) in four cases (33%) and to GAT (aspartic acid) in two cases (17%) (Table 2). In tumours developed in non-smoking subjects no mutations at codon 12 of the K-*ras* gene were observed. The different distribution of *ras* mutations in smoking subjects and non-smoking subjects was statistically significant (Fisher's exact test, $P = 0.002$).

### Associations between genetic alterations and correlations with clinicopathological features

In tumours from non-smoking subjects, LOH affecting microsatellite markers within the FHIT gene and p53 gene mutations were constantly associated (seven cases, see Table 1) (Fisher's exact test, $P < 0.0001$). Conversely, of the eight adenocarcinomas from smoking subjects having a mutated p53 gene, six (75%) did not show microsatellite alterations within the FHIT locus (Table 2). This difference was statistically significant (Fisher's exact test, $P = 0.007$). p53 and K-*ras* mutations in tumours from smoking subjects were not associated, with only one exception. In the group of smoking subjects, p53 mutations were significantly linked with metastatic involvement of thoracic lymph nodes and late-stage disease (contingency table, $P = 0.0182$ and $P = 0.0165$ respectively). A trend was noted towards association between p53 mutations and metastatic spread in the series of tumours from non-smoking subjects, but the data were not significant. No correlations were found between LOH at the FHIT locus or K-*ras* gene alterations and clinicopathological data in lung tumours from smoking subjects.

### DISCUSSION

The genomic status of p53 and K-*ras* genes, and the presence of LOH at the FHIT locus have been investigated in a series of lung adenocarcinomas that developed in subjects who had never smoked. Deletions at chromosomal region 3p14.2 and p53 abnormalities were present in 20% of cases and the alterations were constantly associated. Point mutations of the K-*ras* gene were never observed in this series of lung adenocarcinomas. In lung tumours from smoking subjects the frequency of LOH at the FHIT locus was significantly higher, in agreement with previous results (Sozzi et al, 1997), suggesting that FHIT may be a specific molecular target of carcinogens present in tobacco smoke. The frequency of p53 mutations in smoking adenocarcinomas was similar to that previously reported by other groups in this particular histotype of lung cancer

**Table 2** Genetic alterations and clinicopathological parameters in adenocarcinomas from heavy-smoking subjects

| Cases | FHIT locus | p53 gene | K-*ras* gene | T | N | Stage |
|---|---|---|---|---|---|---|
| 1 | – | – | GAT | T2 | N0 | I |
| 2 | LOH | – | TGT | T2 | N0 | I |
| 3 | – | Codon 175: CGC (Arg) → CTC (Leu) | – | T2 | N2 | III |
| 4 | LOH | – | – | T2 | N0 | I |
| 5 | – | Codon 155: ACC (Thr) → GCC (Ala) | – | T1 | N1 | II |
| 6 | LOH | – | – | T1 | N0 | I |
| 7 | LOH | – | – | T2 | N0 | I |
| 8 | – | – | – | T1 | N1 | II |
| 9 | – | – | – | T2 | N0 | I |
| 10 | – | Codon 249: AGG (Arg) → AGT (Ser) | TGT | T3 | N2 | III |
| 11 | – | – | – | T2 | N0 | I |
| 12 | – | – | – | T2 | N0 | I |
| 13 | – | Codon 171: GAG (Glu) → GGAG (frameshift insertion) | – | T2 | N2 | III |
| 14 | LOH | – | GTT | T2 | N0 | I |
| 15 | LOH | – | – | T2 | N0 | I |
| 16 | LOH | Codon 214: CAT (His) → CGT (Arg) | – | T1 | N0 | I |
| 17 | LOH | – | – | T2 | N1 | II |
| 18 | – | – | – | T2 | N2 | III |
| 19 | LOH | – | – | T2 | N0 | I |
| 20 | LOH | – | – | T3 | N2 | III |
| 21 | LOH | – | – | T2 | N0 | I |
| 22 | – | – | – | T2 | N0 | I |
| 23 | LOH | – | GTT | T2 | N0 | I |
| 24 | – | Codon 216: GTG (Val) → TTG (Leu) | – | T1 | N0 | I |
| 25 | – | – | GAT | T1 | N0 | I |
| 26 | – | – | TGT | T2 | N1 | II |
| 27 | LOH | – | GTT | T2 | N0 | I |
| 28 | – | – | TGT | T1 | N0 | I |
| 29 | LOH | – | – | T2 | N1 | II |
| 30 | LOH | – | TGT | T2 | N0 | I |
| 31 | LOH | – | TGT | T2 | N0 | I |
| 32 | LOH | Codon 245: GGC (Gly) → TGC (Cys) | – | T2 | N2 | III |
| 33 | LOH | – | GGT | T2 | N0 | I |
| 34 | – | Codon 204: GAG (Glu) → TAG (stop) | – | T2 | N0 | I |
| 35 | LOH | – | – | T2 | N0 | I |

© Cancer Research Campaign 1998

**Figure 1** Microsatellite analysis of 35 lung tumours developed in subjects who had never smoked and 35 adenocarcinomas from heavy smokers with three polymorphic markers (*D3S1234*, *D3S1300*, *D3S4103* internal to the FHIT gene. ⬭, Heterozygous; ⬬, LOH; ⊘, not informative

(Li et al, 1994) and not significantly different from that observed in the present series of lung tumours from non-smoking subjects. However, in the group of smoking subjects, we found mostly G:C→T:A transversions, whereas frameshift mutations and G:C→A:T transitions were more frequently seen in tumours from non-smoking subjects. These data, in keeping with results obtained on smaller series of tumours from non-smoking subjects, suggest that endogenous mutational mechanisms, such as DNA polymerase infidelity, deamination of 5-methylcytosine and spontaneous depurination could play a fundamental role in lung carcinogenesis in non-smoking subjects. The frequency and type of codon 12 K-*ras* mutations in the group of adenocarcinomas from patients with a history of smoking was similar to that previously reported in the literature (Rodenhuis and Slebos, 1988; Slebos et al, 1991) and significantly different from that observed in tumours from non-smoking subjects. In conclusion, our data confirm that mutations of the p53 and K-*ras* genes and LOH at the FHIT locus are associated with tobacco smoking; in addition, they indicate that the distribution of such genetic abnormalities in adenocarcinomas from non-smoking subjects is different: k-*ras* mutations are rare events, whereas p53 and FHIT loci are concomitantly altered in 20% of cases.

The constant association of LOH at 3p14.2 and p53 abnormalities in tumours from non-smoking subjects is intriguing. Anamnestical data were carefully collected; therefore, in the non-smoking subjects' group we can exclude a history of smoking, even limited to a small number of cigarettes for few years. However, in 75% of cases an exposition to environmental tobacco smoke was documented (Table 1). A number of considerations let us conclude that the observed association of p53 gene abnormalities and LOH at the FHIT locus is independent from the effect of environmental tobacco smoking: (a) no significant association was present between the exposure of the patient to environmental smoking and these two genetic changes; (b) in the group of patients with a history of smoking, the frequency of cases with concomitant alteration of the FHIT locus and p53 gene was significantly lower than that observed in non-smoking subjects; (c) p53 alterations in tumours from non-smoking subjects were mostly G:C→A:T transitions and deletions; (d) no mutations of the K-*ras* gene at codon 12, which is known to

© Cancer Research Campaign 1998

be a specific target of the mutagenic activity of tobacco smoke, were found in tumours showing FHIT and p53 abnormalities.

Alterations of a gene(s) involved in DNA mismatch repair could lead to genetic instability and explain the concomitant presence of gene defects (Aaltonen et al, 1993). As we did not find a microsatellite instability outside of the FHIT locus in tumours with FHIT deletions, the observed association of p53 and FHIT aberrations does not seem to be ascribable to mismatch repair gene deficiency leading to replication errors (RERs). Another possibility is that abnormalities in the p53 gene itself may destabilize the genome, favouring the presence of multiple genetic anomalies (Livingstone et al, 1992; Yin et al, 1992). In keeping with this hypothesis, it has been recently observed that 3p14 deletions are more frequent in cervical carcinomas associated with papillomavirus infection and p53 inactivation (Boldog et al, 1997). On the other hand, p53 mutations appear uncommon in RER+ colorectal carcinomas and gastric tumours (Wu et al, 1994; Renault et al, 1996). In the light of these observations, our results suggest that in tumours from non-smoking subjects LOH at the FHIT locus are not a consequence of mismatch repair deficiency, but may be related with the genomic instability that accompanies p53 mutations. However, a concomitant association of 3p14.2 deletions and p53 gene abnormalities was not frequent in tumours from heavy smoking subjects, indicating that this association is not a constant event in human cancer. In particular, in tumours from smoking subjects, FHIT gene alterations may be primarily related to carcinogens present in tobacco smoke and not a consequence of p53 inactivation.

In the series of smoking patients p53 mutations were significantly associated with metastatic involvement of hilar/mediastinal lymph nodes and advanced stages of disease, in agreement with previous results (Marchetti et al, 1993; Lee et al, 1994). In non-smoking subjects a trend towards these associations was observed, but the data did not reach statistically significant values.

In conclusion, our results indicate that lung tumours developed in never-smoking subjects represent a distinct biological entity in which LOH at the FHIT locus and p53 mutations are concomitantly present in 20% of cases. On the contrary, this association of molecular events was uncommon in tumours from heavy-smoking subjects. As different p53 mutations were observed in these two groups of lung tumours, we are tempted to hypothesize that LOH at the FHIT locus in never-smoking subjects' lung cancer may be dependent from the particular type of p53 mutations (G:C→A:T transitions and deletions). At this point it should be interesting to evaluate the status of FHIT and p53 in other forms of human malignancies in order to assess whether the association of genetic abnormalities observed is restricted to lung cancer in non-smoking subjects or common to other forms of human neoplasms.

### ACKNOWLEDGEMENTS

This work was supported by: CNR target project ACRO N. 96.00591.PF39; AIRC, Italian Association for Cancer Research; MURST 40%. Silvia Pellegrini is supported by a fellowship from AIRC.

### REFERENCES

Aaltonen LA, Peltomaki P, Leach FS, Sistonen P, Pylkkanen L, Mecklin J-P, Jarvinen H, Powell SM, Jen J, Hamilton SR, Petersen GM, Kinzler KW, Vogelstein B and De La Chapelle A (1993) Clues to the pathogenesis of familial colorectal cancer. *Science* **260**: 812–816

Blin N and Stafford DW (1976) A general method for isolation of high molecular weight DNA from eucaryotes. *Nucleic Acid Res* **3**: 2303–2308

Boldog F, Gemmil R M, West J, Robinson M, Robinson L, Efang L, Roche J, Todd S, Waggoner B, Lundstrom R, Jacobson J, Mullokandov MR, Klinger H and Drabkin H A (1997) Chromosome 3p14 homozygous deletions and sequence analysis of FRA3B. *Hum Mol Gen* **6**: 193–203

Brownson RC, Loy TS, Ingram E, Myers JL, Alavanja MCR, Sharp DJ and Chang JC (1995) Lung cancer in nonsmoking women. *Cancer* **75**: 29–33

Denissenko MF, Pao A, Tang M and Pfeifer GP (1996) Preferential formation of benzo(a)pyrene adducts at lung cancer mutational hotspots in p53. *Science* **274**: 430–434

Lee LN, Shew JY, Sheu JC, Lee YC, Lee WC, Fang MT, Chang HF, Yu CJ, Yang PC and Luh KT (1994) Exon 8 mutations of p53 gene associated with nodal metastasis in non-small-cell lung cancer. *Am J Resp Crit Care Med* **150**: 1667–1671

Li ZH, Zheng J, Weiss LM, Shibata D (1994) c-K-ras and p53 mutations occur very early in adenocarcinoma of the lung. *Am J Pathol* **144**: 303–309

Livingstone LR, White A, Sprouse J, Livanos E, Jacks T and Tlsty TD (1992) Altered cell cycle arrest and gene amplification potential accompany loss of wild type p53. *Cell* **70**: 923–935

Marchetti A, Buttitta F, Merlo G, Diella F, Pellegrini S, Pepe S, Macchiarini P, Chella A, Angeletti CA, Callahan R, Bistocchi M, Squartini F (1993) *p53* alterations in non-small cell lung cancer correlate with metastatic involvement of hilar and mediastinal lymph-nodes. *Cancer Res* **53**: 2846–2851

Marchetti A, Buttitta F, Pellegrini S, Chella A, Bertacca G, Filardo A, Tognoni V, Ferreli F, Signorini E, Angeletti CA and Bevilacqua G (1996) K-ras mutations in bronchioloalveolar lung carcinomas: a constant event in the mucinous subtype. *J Pathol* **179**: 254–259

Mountain CF (1986) A new international staging system for lung cancer. *Chest* **89**: 225S–233S

Renault B, Calistri D, Buonsanti G, Nanni O, Amadori D and Ranzani, GN (1996) Microsatellite instability and mutations of p53 and TGF-beta RII genes in gastric cancer. *Hum Genet* **98**: 601–607

Rodenhuis S and Slebos RJC (1988) Incidence and possible clinical significance of ras oncogene activation in adenocarcinomas of the human lung. *Cancer Res* **48**: 5738–5741

Shopland DR, Eyre HJ and Pechacek TF (1991) Smoking-attributable cancer mortality in 1991: is lung cancer now the leading cause of death among smokers in the United States? *J Natl Cancer Inst* **83**: 1142–1148

Slebos RJ, Hruban RH, Dalesio O, Mooi WJ, Offerhaus GJ and Rodenhuis S (1991) Relationship between K-ras oncogene activation and smoking in adenocarcinomas of the human lung. *J Natl Cancer Inst* **83**: 1024–1027

Sozzi G, Veronese ML, Negrini M, Baffa R, Cotticelli MG, Inoue H, Tornielli S, Pilotti S, De Gregorio L, Pastorino U, Pierotti MA, Ohta M, Huebner K and Croce CM (1996) The FHIT gene at 3p14.2 is abnormal in lung cancer. *Cell* **85**: 17–26

Sozzi G, Sard L, De Gregorio L, Marchetti A, Musso K, Buttitta F, Tornielli S, Pellegrini S, Veronese ML, Manenti G, Incarbone M, Chella A, Angeletti CA, Pastorino U, Huebner K, Bevilacqua G, Pilotti S, Croce CM and Pierotti MA (1997) Association between cigarette smoking and FHIT alterations in lung cancer. *Cancer Res* **57**: 1121–1123

Suzuki H, Takahashi T, Kuroishi T, Suyama M, Ariyoshi Y and Hueda R (1992) p53 mutations in non-small cell lung cancer in Japan: association between mutations and smoking. *Cancer Res* **52**: 734–736

Takahashi T, Nau MM, Chiba I, Birrer MJ, Rosenberg RK, Vinocour M, Levitt M, Pass H, Gazdar AF and Minna JD (1989) p53: a frequent target for genetic abnormalities in lung cancer. *Science* **246**: 491–494

Takeshima Y, Seyama T, Bennet WP, Akiyama M, Tokuoka S, Inai K, Mabuchi K, Land CE and Harris CC (1993) p53 mutations in lung cancers from non-smoking atomic-bomb survivors. *Lancet* **342**: 1520–1521

World Health Organization (1982) The World Health Organization. Histological typing of lung tumours. *Am J Clin Pathol* **77**: 123–136

Wu C, Akiyama Y, Miyake S, Nagasaki H, Oto M, Okabe S, Iwama T, Mitamura K and Masumitsu H (1994) DNA alterations in cells from hereditary non-polyposis colorectal cancer patients. *Oncogene* **9**: 991–994

Yin Y, Tainsky MA, Bischoff FZ, Strong LC and Wahl GM (1992) Wild-type p53 restores cell cycle control and inhibits gene amplification in cells with mutant p53 alleles. *Cell* **70**: 937–948

© Cancer Research Campaign 1998